# Multiple Introductions of Multidrug-Resistant Tuberculosis into Households, Lima, Peru

**DOI:** 10.3201/eid1706.101471

**Published:** 2011-06

**Authors:** Ted Cohen, Megan Murray, Ibrahim Abubakar, Zibiao Zhang, Alexander Sloutsky, Fernando Arteaga, Katiuska Chalco, Molly F. Franke, Mercedes C. Becerra

**Affiliations:** Author affiliations: Brigham and Women’s Hospital, Boston, Massachusetts, USA (T. Cohen, M. Murray, Z. Zhang, M.C. Becerra); Harvard School of Public Health, Boston (T. Cohen, M. Murray); University of East Anglia, Norwich, UK (I. Abubakar); Health Protection Agency Centre for Infections, London, UK (I. Abubakar); University of Massachusetts Medical School, Boston (A. Sloutsky); Partners In Health, Boston and Lima, Peru (F. Arteaga, K. Chalco, M.F. Franke, M.C. Becerra); and Harvard Medical School, Boston (M.F. Franke, M.C. Becerra)

**Keywords:** antimicrobial resistance, multidrug-resistant tuberculosis, MDR TB, bacteria, tuberculosis and other mycobacteria, transmission, genotype, household, Peru, research

## Abstract

Two cases of multidrug-resistant tuberculosis (MDR TB) in a household are assumed to reflect within-household transmission. However, in high-incidence areas of MDR TB, secondary cases may arise through exposure to MDR TB in the community. To estimate the frequency of multiple introductions of MDR TB into households, we used spoligotyping and 24-loci mycobacterial interspersed repetitive unit–variable number tandem repeats to classify isolates from 101 households in Lima, Peru, in which >1 MDR TB patient received treatment during 1996–2004. We found different MDR TB strains in >10% of households. Alternate approaches for classifying matching strains produced estimates of multiple introductions in <38% of households. At least 4% of MDR TB patients were reinfected by a second strain of MDR *Mycobacterium tuberculosis*. These findings suggest that community exposure to MDR TB in Lima occurs frequently. Rapid drug sensitivity testing of strains from household contacts of known MDR TB patients is needed to identify optimal treatment regimens.

## Abstract 2

Data on household transmission are needed to develop optimal treatment.

The discovery and use of discriminating genetic markers such as IS*6110* restriction fragment length polymorphisms (RFLPs), spacer oligonucleotides (spoligotyping), and mycobacterial interspersed repetitive unit–variable number tandem repeats (MIRU-VNTRs) (*1*) have improved our understanding of the transmission dynamics of tuberculosis (TB) (*2**,**3*). Genotyping studies, in which strains with matching sets of markers are considered potential members of a single transmission chain, have demonstrated that recent transmission plays a major role, even in low-incidence settings (*4**,**5*); that persons with recurrent episodes of TB may be having reinfection rather than relapse (*6**–**8*); that persons may be infected by >1 isolate of *Mycobacterium tuberculosis* at the same time (*9**–**11*); and that transmission may occur in casual social settings (*12*).

Molecular epidemiologic studies have also demonstrated that secondary cases among close associates of known case-patients are not always members of the same chain of transmission, i.e., that infection may have been acquired from independent sources (*13*). Molecular investigations of households of multiple TB patients showed that cohabitating TB patients may be infected with distinct isolates of *M*. *tuberculosis* (*14**–**16*). For example, in 2 suburbs of Cape Town, South Africa, which have TB notification rates of ≈320 cases per 100,000 population, researchers found that less than half (46%) of secondary TB cases within households had a TB isolate that matched an isolate from another case within the household by RFLP (*16*). Overall, <1 (19%) in 5 new TB cases occurring in these communities was the result of within-household transmission.

Although studies have shown that household contacts with TB are likely to have acquired infection independently in high-incidence settings, there are no published estimates of the probability that 2 household members with multidrug-resistant TB (MDR TB: resistance to at least isoniazid and rifampin) share a similar genotype and are members of the same transmission chain. Molecular epidemiologic data from households with >1 MDR TB case can help shed light on the transmissibility of highly drug-resistant disease and also help guide public health policy. For example, international guidelines for the management of known contacts of MDR TB patients recommend an empirical drug regimen based either on the drug-resistance profile of an isolate from the suspected index MDR TB case-patient or on the most common drug-resistance pattern in the community while drug sensitivity tests are pending (*17**–**19*). A better understanding of the relative importance of intrahousehold or community transmission may help to inform the choice of empirical regimen.

Despite a decreasing overall incidence of TB in Peru of ≈3.7% per year since 1996, the incidence of MDR TB has increased by ≈4.5% over the same period (*20*). The increasing incidence of MDR TB in densely occupied urban communities of Lima, Peru, poses obvious challenges for TB control. We report a molecular epidemiologic study within households in Lima in which >1 person received a diagnosis of MDR TB. We used spoligotyping and 24-loci MIRU-VNTR typing (*21**,**22*) to identify households that have had >1 introduction of MDR TB, and we explored the association of household factors with these multiple introduction events.

## Materials and Methods

### Study Setting, Participants, and Data

The estimated incidence of TB in Lima, Peru, is >130 cases/100,000 persons; this estimate masks substantial heterogeneity in the actual distribution of TB within this large metropolitan area where poor areas often experience several-fold higher local incidence of disease than higher-income areas (*23*). For example, in 2000 in northern metropolitan Lima (population 3,186,199), the incidence of active TB was 232 cases/100,000 persons (*24*). A nationwide survey in 2006 reported that 5.3% of all new cases and 23.6% of retreatment cases were MDR TB (*25*). Since 1996, Partners in Health and Socios en Salud Sucursal Peru have worked with the Peru Ministry of Health to implement a program to treat patients with active MDR TB by using supervised, individualized, antimicrobial drug regimens delivered on an ambulatory basis (*26**–**28*).

We previously reported the TB incidence in a cohort of household contacts of the patients treated for MDR TB (*29*). A household was eligible for inclusion in the study if >2 members had been treated for MDR TB by this program during 1996–2004, and if >1 MDR *M. tuberculosis* isolate obtained from each person was available for analysis. All available (pretreatment and ongoing treatment) MDR isolates from patients in eligible households were included in this analysis. Demographic data, drug-susceptibility test results, and information about the physical condition of the household structure were abstracted from the electronic records of the MDR TB program. This study was reviewed and approved by the Committee on Human Studies of the Office of Research Subject Protection of Harvard Medical School.

### Laboratory Methods and Drug-Susceptibility Testing

Drug-susceptibility testing and genotyping by using MIRU-VNTR and spoligotyping were performed by the Supranational Reference Laboratory at the University of Massachusetts Medical School. A standard agar plate proportion method was used for drug-susceptibility testing of *M. tuberculosis* isolates. The first-line and second-line drugs tested were isoniazid (0.2 mg/L, 1.0 mg/L, and 5.0 mg/L), rifampin (1.0 mg/L), streptomycin (2.0 mg/L and 10.0 mg/L), ethambutol (5.0 mg/L), kanamycin (5.0 mg/L), ethionamide (10.0 mg/L), capreomycin (10.0 mg/L), ofloxacin (2.0 mg/L), and *p*-amino salicylic acid (8.0 mg/L). Susceptibility to pyrazinamide (100 mg/L) was determined by using the BACTEC 460 Liquid Medium System (Becton Dickinson, Sparks, MD, USA). We only included drugs to which resistance had been tested for >70% of isolates in the study.

### MIRU-VNTR Genotyping

DNA for PCR analysis was prepared by using a simple thermolysis procedure. PCR amplification of the 24 MIRU-VNTR loci was conducted as described (*22**,**30*) with minor modifications. The PCR mixture contained 2 µL of thermolysate, 1× PCR buffer, 1 mol/L betaine, 0.5 U Taq DNA polymerase (Takara Bio, Madison, WI, USA), 200 µmol/L of each dNTP, and 0.3 µmol/L of each flanking primer.

An ABI Thermal Cycler 2720 (Applied Biosystems, Foster City, CA, USA) was used for PCRs. Initial denaturation at 94°C for 5 min was followed by 35 cycles of denaturation at 94°C for 30 s, annealing at 62°C for 30 s, and elongation at 70°C for 45 s; and a final extension step at 72°C for 10 min. *M. tuberculosis* H37RV DNA and sterile distilled water were included in each test run as positive and negative controls, respectively.

PCR products were analyzed in 2 ways. First, DNA fragments from amplification with primers specific for loci ETRA, ETRB, ETRC, ETRD, MIRU2, MIRU20, MIRU23, MIRU24, MIRU26, Mtub21, Mtub29, Mtub30, Mtub34, and Qub11b were separated by using standard 2% agarose gel electrophoresis. Second, DNA fragments from amplification with primers specific for loci ETRE, MIRU10, MIRU16, MIRU27, MIRU39, MIRU40, Mtub04, Mtub39, Qub26, and Qub4156 were analyzed by electrophoresis with the QIAxcel System and the QIAxcel DNA Screening Kit (both from QIAGEN, Valencia, CA, USA).

### Spoligotyping

Mycobacterial DNA was prepared by using the same thermolysis protocol as for MIRU-VNTR typing. For DNA amplification, 0.15 µL *Tth* polymerase (5 U/µL; Roche, Pleasanton, CA, USA) was added to 50 µL of PCR mixture, and the following amplification profile was used: 3 min at 96°C; 35 cycles for 1 min at 96°C, 1 min at 55°C, and 30 s at 72°C; and 5 min at 72°C.

Spacer oligonucleotide typing was performed by using the Multianalyte Profiling System (Luminex Inc., Austin, TX, USA). The procedure was conducted according to the protocol reported by Cowan et al. (*31*) with adaptations for a 96-well format. Fluorescence signals indicating hybridization strength were analyzed by using Bio-Plex Suspension Array System Instrument Luminex 100xMAP Technology (Luminex Molecular Diagnostics Inc., Toronto, Ontario, Canada) and the Bio-Rad BioPlex Manager Program version 4.1.1 (Bio-Rad Laboratories, Hercules, CA, USA). Lineage and the shared type for each isolate were assigned based on matching the spoligotype patterns with those listed in the SpolDB4 database (*32*).

### Identification of Multiple Introductions of *M. tuberculosis* into a Household

Households were classified as having evidence of repeated introduction of TB from the community if isolates from >2 patients with MDR TB within 1 household had different molecular genotypes. Supply et al. proposed a standard approach for characterizing the relatedness of *M. tuberculosis* isolates by spoligotyping and 24-loci MIRU-VNTR. They found that the combination of these methods (which requires including >15 of the most diverse loci for MIRU-VNTR analysis) has comparable discriminatory power to IS*6110* RFLP typing (*22*). We present minimum and maximum estimates of the proportion of households judged to have evidence of multiple TB introductions on the basis of spoligotyping and MIRU-VNTR genotyping data.

We also examined a classification approach recently used by Narayanan et al. (*7*). Nonmatching strains are defined as those strains with >1 spoligotype spacer or >1 MIRU-VNTR locus difference. Enabling different degrees of stringency in calling 2 (or more) strains a match reflects our underlying uncertainty about how rapidly spoligotypes and MIRU-VNTR genotypes change because of mutations at marker loci during the natural history of disease and through chains of transmission that may span decades.

### Identification of Reinfection Events

We genotyped all available MDR isolates of patients within study households. Among participants from whom >2 isolates were available, we identified episodes of reinfection on the basis of differences in genotypes. We used a similar approach for comparing genotypes for identifying episodes of reinfection and repeated household introduction.

### Statistical Analysis

SAS version 9.2 (SAS, Cary, NC, USA) was used for statistical analysis. We performed standard nonparametric tests for assessing univariate associations between household-level factors and the probability of repeated introduction.

## Results

We identified 105 households in which >1 MDR *M. tuberculosis* isolate was available from each of >2 different household members. In total, 391 MDR isolates from 236 persons were available for molecular typing. Spoligotyping and MIRU-VNTR analyses were successfully completed on samples from >2 participants from 101 (96%) of these households. These analyses resulted in a set of 384 (98%) isolates from 232 (98%) persons. Characteristics of persons and households included in the study are shown in Table 1. There were an additional 142 households for which we knew of >2 patients with MDR TB, but for whom *M. tuberculosis* specimens were no longer available for genetic analysis. No statistically significant differences in size, density, or age distribution of members were found between the households that were included and those not included in this study.

**Table 1:** Characteristics of 101 households with MDR TB, Lima, Peru, 1996–2004*

Characteristic	Median (IQR)
Persons per household	8 (6–10)
Persons per bedroom	2.5 (1.75–4.33)
Participants per household	2 (2–2)
Participants, n = 232	
Age, y	23.8 (19.2–30.5)
Male sex, %	57.2

*MDR TB, multidrug-resistant tuberculosis; IQR, interquartile range.

Of 384 isolates, 228 (59%) were tested for susceptibility to a sufficient number of second-line drugs to identify extensively drug-resistant *M. tuberculosis* strains (MDR plus additional resistance to a fluoroquinolone and a second-line, injectable antimicrobial drug [either kanamycin, amikacin, or capreomycin]). Thirty-one (14%) of these 228 isolates were confirmed as extensively drug resistant and were obtained from 15 patients, none of whom were living in the same household.

### Multiple Introductions of MDR *M. tuberculosis* into Households

Using a permissive definition of matching in which we included strains that differed by 1 spoligotype spacer to be matched, we estimated that 10 (10%) of households had distinct MDR isolates and showed evidence of repeated introduction. The strictest definition of matching, which required exact matches in spoligotype and at all 24-loci of the MIRU-VNTR analysis, showed that 38 (38%) of households had evidence of repeat introduction of MDR TB from the community (Figure). Using the approach of Narayanan et al. (*7*) for identifying nonmatching strains (pairs with >1 spoligotype spacer or 1 MIRU-VNTR locus difference), we classified 16 (16%) households as settings with multiple introductions of MDR TB.

**Figure:**
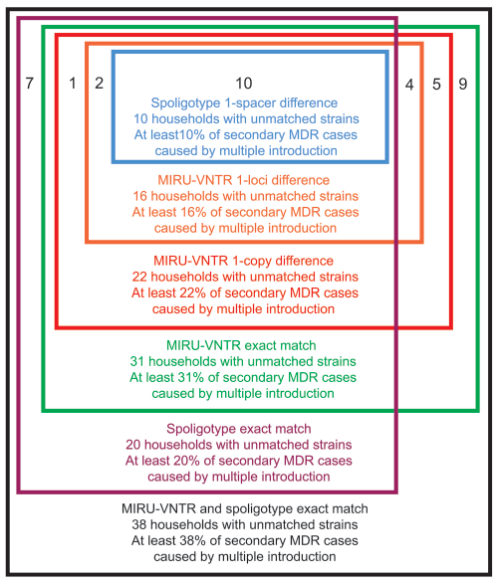
Numbers of households classified as having multiple multidrug-resistant (MDR) tuberculosis introductions by 6 definitions of matching genotypes, Lima, Peru, 1996–2004. MIRU-VNTR, mycobacterial interspersed repetitive unit–variable number tandem repeat.

The 16 households in which >2 persons had an MDR *M. tuberculosis* isolate that was different from that obtained from another person in the household, according to the definition of Narayanan et al. (*7*), are shown in Table A1. Seven of these households also had evidence of within-household transmission of MDR TB. Closer inspection of spoligotypes isolated from these households indicated that 6 of the 16 households, although failing to meet the proposed criterion for matching, had similar isolates (households 112, 192, 557, 960, 263, and 645). If these 6 households are classified as having evidence of within-household transmission, our best estimate of the number of households with evidence of multiple introductions of MDR strains is reduced to 10 (10%). Under these criteria, the percentage of households with only evidence of probable within-household transmission is 90%.

We used the 10 households as our most conservative set of households with evidence of multiple introductions of MDR stains and searched for household factors that were associated with multiple introduction events. We did not find any significant associations; specifically, the size and density of households, the quality of the household structure, and time span over which isolates were accrued from households all appeared to be unrelated to multiple introductions (Table 2). In addition, no significant difference was found in the number of drugs to which the isolate from the first patient was resistant between households that had repeated introduction (mean 5.1 drugs) and households that had evidence of probable within-household transmission (mean 5.3 drugs; p = 0.75).

**Table 2:** Association between household factors and repeated introduction of MDR TB, Lima, Peru, 1996–2004*

Factor	Introduction, n = 10†	No introduction, n = 91	p value
No. persons	7.5 (6–8)	8 (7–11)	0.18
Persons per bedroom	2.6 (1.7–2.7)	2.4 (1.75–5)	0.43
Homes of substandard quality‡	1/9 (11)	23/64 (36)	0.44
Mean age of household members, y	28 (23–32)	26 (21–30)	0.39
Duration between first and last isolate obtained from household, d	389 (167–724)	345 (204–599)	0.92

*Values are median (interquartile range) or no. positive/no. tested (%). MDR TB, multidrug-resistant tuberculosis. †Households classified as having repeated MDR TB introductions for these analyses are indicated in Table A1 (www.cdc.gov/EID/content/17/6/969-appT1.htm). ‡Substandard housing was defined as a dwelling with a dirt floor; walls made of straw matting, plastic, or plywood; a roof made of straw matting, plastic, or plywood; or no access to water in the home (data were not available for all households).

### Evidence of MDR Reinfection

Ninety persons had >1 MDR TB isolate available for analysis. Using the definition of matching strains of Narayanan et al. (*7*), we found that 5 (6%) of these persons had 2 distinct strains of MDR *M*. *tuberculosis* during the period of follow-up and the remaining 85 (94%) showed repeated isolation of the same MDR strain (Table A2). Closer inspection of the isolates available from these 5 persons showed that 1 person (a 20-year-old man) from household 977 may not have been reinfected. Three isolates were available from this person. The first isolate had a slightly different spoligotype than the 2 isolates subsequently obtained, but the MIRU-VNTR pattern was the same for all 3 isolates.

## Discussion

In the absence of molecular epidemiologic data, secondary cases of MDR TB within a household are generally assumed to be the result of within-household transmission. In an area with increasing incidence of MDR TB (*20*), we found that 90% of household contacts of MDR TB index cases with active disease and drug-susceptibility test results had MDR TB (*29*). Our present study, in a subset of that cohort, used genotyping on the basis of spoligotyping and 24-loci MIRU-VNTR, which has been shown in other settings to have comparable discriminatory power to IS*6110* RFLP (*21*). Our study shows that there was at least a 10% risk that a subsequent case of MDR TB occurring within the home of a known MDR TB patient was the result of transmission in the community rather than transmission in the household. This estimate represents a lower boundary of the contribution of community transmission to the appearance of secondary MDR cases within a home because matching strains within a household (which we would categorize as within-home transmission) may be caused by transmission from other sources in the community. Because circulating MDR strains were heterogeneous (Table 3), the magnitude of this bias may not be substantial.

**Table 3:** Strain lineages of *Mycobacterium tuberculosis* detected in the study population, Lima, Peru, 1996–2004

Lineage	No. (%)
Beijing	19 (4.9)
H1	22 (5.7)
H3	22 (5.7)
LAM1	25 (6.5)
LAM3	12 (3.1)
LAM4	6 (1.6)
LAM5	47 (12.2)
LAM9	38 (9.9)
T1	85 (22.1)
T2	19 (4.9)
T5_MAD2	2 (0.5)
U	1 (0.3)
X3	17 (4.4)
No match	69 (18.0)

We did not find any easily measured household factors associated with risk for repeated introductions compared with within-home transmission. We had hypothesized that a high household density (persons/bedroom) or low quality of household structure may be associated with a higher probability of within-home transmission, conditional upon observing multiple cases within a home, but this hypothesis was not supported by these data. This finding may reflect an absence of this association between household characteristics and risk for within-home transmission or, alternatively, it may reflect the relatively small number of repeated introduction events that we observed and our limited power to test such associations. Accordingly, although our observations provide convincing evidence that repeated introduction of MDR TB into households occurs in these settings, further studies are needed to determine whether household factors, number of persons within these households, or strains present within these households are associated with an increased risk for within-home transmission or repeated exposure in the community.

Genetic (*33*) or acquired susceptibility (*34*) to infection and disease may play a role in the accumulation of multiple TB cases within households. Because household members are likely to share genetic or environmental risk factors, or both, persons living with TB case-patients may be particularly likely to be infected and acquire disease whether they are infected by their household contact or in the community.

Our findings provide evidence to support international guidelines for management of active TB among contacts of known MDR TB cases (*17**–**19*) because they confirm that among strains from persons for which genotyping test results are available, <90% of household contacts with MDR TB were infected with the same strain as the index patient. Our findings also highlight limitations associated with such policies. Because subsequent cases of MDR TB in a household may be caused by community transmission, policies that specify that apparent secondary case-patients receive therapy on the basis of the drug-susceptibility profile of an isolate from the initial MDR TB patient may result either in effective drugs being needlessly withheld or in administration of drugs to which the strain is already resistant. This policy may result in acquisition of additional resistance to second-line drugs and prolonged opportunity for transmission of highly drug-resistant strains within homes and in the community (*35**,**36*).

These findings support the use of rapid drug-resistance tests to determine drug susceptibility profiles in known contacts of MDR TB patients. Molecular tests for resistance, such as line probe assays and cartridge-based PCRs (i.e., GeneXpert; Cepheid, Sunnyvale, CA, USA), are promising and have been endorsed by the World Health Organization for determining resistance to first-line drugs (*37*). However, although new diagnostic tests in development also detect resistance to second-line drugs (*38**,**39*), these tests have not yet been optimized for use in guiding clinical care. New rapid phenotypic tests for resistance, such as the microscopic-observation drug-susceptibility assay, have also not yet been adequately tested under field conditions for their capacity to be used in selection of tailored regimens for MDR TB (*40*). Known contacts of MDR TB patients should be a high-priority, high-yield study population for assessing the immediate utility of these new tools.

A limitation of our study is that we cannot definitively distinguish the 2 mechanisms by which distinct MDR isolates may appear within households. First, household members may have been infected by different drug-susceptible strains in the community and acquired drug resistance through deficient drug treatment. Second, household members may have been directly infected by different MDR strains in the community. Distinguishing between these 2 possibilities is essential because each would cause a distinct public health response. The first mechanism suggests that detailed investigation of individual-level or household-level risk factors for acquisition of MDR TB was needed and would indicate a need for greater treatment support and supervision for patients with drug-susceptible disease. The second mechanism indicates a need to improve infection control in the community or to facilitate diagnosis and effective treatment for persons with MDR TB to reduce the duration of infectiousness. In most circumstances, we expect acquisition and transmission to contribute to the appearance of multiple cases of MDR TB within homes, and efforts to reduce the incidence of drug-resistant disease will need to address these factors.

Although we have insufficient data for previous TB episodes and treatment for persons in our study to exclude possible independent acquisition of MDR TB among household members because of inadequate treatment, our finding that >4 persons showed evidence of reinfection by a second (i.e., different) MDR TB strain provides evidence that there is a high risk for MDR TB exposure in this community. HIV status was known for only ≈50% of the persons in the study. Among those tested, only 3 (3%) of 102 were HIV infected and none of the 3 HIV-infected persons were among persons in households in which multiple introductions of MDR TB were detected. If co-infection with HIV was common, it would be expected to increase the probability of rapid progression to disease and lead to higher risks of multiple cases of unlinked disease within households. Because HIV co-infection was so rare, it is unlikely that this explains the study results.

Our results extend findings from previous studies showing that a substantial fraction of cohabiting persons have independently acquired TB in the community (*13**–**16*). In contrast to earlier studies that compared relative contributions of within-home and community transmission, all persons in our study had MDR TB. We found that although 90% of households had evidence of intrahousehold transmission, 10% had >2 independent introductions of MDR *M. tuberculosis* strains from the community. This finding suggests that the risk for community or extrahousehold transmission of MDR TB in Lima is high. Furthermore, it indicates that known MDR TB contacts initiating empirical treatment for MDR TB treatment require access to drug susceptibility testing to ensure that they receive the drugs to which their isolate is susceptible. National TB programs should be wary of applying empirical regimens on the basis of population-level drug susceptibility data without better understanding of the relative role of intrahousehold and community transmission of MDR TB.

## Appendix

**Table A1:** Households with potential multiple introductions of MDR TB, Lima, Peru, 1996–2004*

House ID no.	Patient age, y/sex	Date	MIRU-VNTR pattern	Spoligotype	ST	Lineage	Drug susceptibility, SHRECFYTKZALP†
4‡	21/M	1997 Feb 1	2 2 2 3 3 2 4 2 2 6 1 5 3 2 3 3 3 4 2 1 2 3 + 2	736177607700171	1476	U	SRRSSSSSSSXXX
	22/M	1997 Jan 14	3 2 3 3 3 2 4 3 1 5 1 5 3 2 4 4 3 4 2 3 4 3 6 3	777740777760771	219	T1	SRRXSSSSSRXXX
112	19/M	2001 Nov 14	4 2 4 3 2 2 4 3 2 1 1 4 3 2 4 3 3 4 2 3 4 3 2 2	777777777760771	53	T1	RRRRRSSSRSRSS
	25/M	2001 Nov 13	4 2 4 3 5 2 3 3 2 1 1 4 3 2 4 3 3 4 2 3 4 3 2 2	777777777760771	53	T1	RRRRRSSRRRRSS
126‡	27/M	2001 May 7	2 1 4 3 3 2 4 2 2 6 1 5 3 2 1 5 2 4 1 5 3 4 8 2	777777607760771	42	LAM9	RRRRSSSRSRSSS
	22/F	2001 May 2	2 1 2 3 2 1 4 3 2 7 1 5 3 2 8 4 2 4 1 4 3 1 5 2	777400007760771	1594	T1	RRRRSRSRSRSRS
192	29/F	2000 Feb 25	2 1 5 3 3 2 3 1 2 4 1 2 2 1 1 5 3 4 1 5 3 3 8 2	777777607760731	60	LAM4	RRRRSRSSSRXXX
	23/F	2001 May 31	2 1 5 3 3 2 3 1 2 4 1 2 2 2 1 4 3 4 1 5 3 3 8 2	777777607760731	60	LAM4	RRRRSSSRSRXSS
361‡	27/M	1998 May 20	3 2 3 2 3 2 5 3 2 5 1 4 3 2 2 5 3 4 1 3 4 5 4 3	700036777760771	91	X3	RRRRXXXXXRXXX
	26/F	1999 Mar 1	4 2 4 3 2 2 3 3 2 1 1 5 3 2 4 3 3 4 2 3 4 3 2 2	777777777760771	53	T1	XRRSSSSSSRXXX
517‡	66/F	2002 May 7	3 2 3 3 3 2 4 3 2 5 1 4 3 2 2 3 4 4 4 3 4 6 6 3	700036777760771	91	X3	RRRRSSSSSRSSS
	30/M	2001 Nov 21	4 2 3 3 3 2 3 3 2 1 1 5 3 2 4 3 3 4 2 3 4 3 2 2	777777777760771	53	T1	RRRSSSSSSSSSS
557	24/M	2003 Apr 25	3 2 3 3 3 2 5 3 2 5 1 5 3 2 3 3 2 4 4 3 5 4 7 3	777777777720761	ND	ND	RRRRSSSRRSRSS
	18/M	2002 Oct 9	3 2 3 3 3 2 5 3 2 5 1 5 3 2 3 3 2 4 4 3 4 5 7 3	777777777720771	50	H3	SRRRSSSRSSSSS
685‡	19/F	2003 Apr 4	3 2 3 3 3 2 5 3 2 5 1 5 3 2 3 3 2 4 4 3 6 5 7 3	777777707720771	512	H3	SRRRSSSSSRSSS
	30/M	2003 Jul 17	4 2 4 3 2 2 3 3 2 1 1 5 3 2 4 3 3 4 2 3 4 3 2 2	777777777760771	53	T1	SRRRSSSSSRSSS
960	19/F	2002 Jul 18	1 1 4 3 3 2 4 2 2 6 1 5 3 2 1 4 3 4 1 5 3 4 4 2	777737607760771	93	LAM5	RRRRSSSSSRSSS
	16/M	2000 Aug 7	2 1 4 3 3 2 4 2 2 6 1 5 3 2 1 4 3 4 1 4 3 4 4 2	777737607760771	93	LAM5	RRRRSSSSSRXXX
28‡	18/M	2001 Feb 20	2 2 4 3 2 1 4 2 2 6 1 5 3 2 2 4 3 4 1 3 3 2 7 2	777774077560771	222	T1	SRRRSSSRSRXSS
	NA	2002 May 25	4 2 4 3 2 2 3 3 2 1 1 5 3 2 4 3 3 4 2 3 4 3 2 2	777777777760771	53	T1	RRRRSSSSSRSSS
	20/M	2001 Aug 31	2 2 4 3 2 1 4 2 2 6 1 5 3 2 2 4 3 4 1 3 3 2 7 2	777774077560771	222	T1	SRRSSSSRSRSSS
106‡	35/M	1997 Aug 23	2 1 4 3 3 2 4 2 2 6 1 5 3 2 1 4 3 4 1 5 3 4 4 2	777737607760771	93	LAM5	SRRRSSSSSRXXX
	33/F	2000 May 5	2 2 + 3 2 1 4 3 2 6 1 4 3 2 0 4 3 4 1 3 3 2 6 2	777777607760771	42	LAM9	RRRRSRSSSRXXX
	15/M	2000 Aug 29	2 2 + 3 2 1 4 3 2 6 1 4 3 2 0 4 3 4 1 3 3 2 6 2	777777607760771	42	LAM9	RRRSSSSSSRXXX
263	75/F	2002 Aug 7	3 2 3 3 3 2 5 3 2 5 1 4 3 2 2 8 2 2 1 3 4 4 6 3	700036777760771	91	X3	RRRRSSSRSRSSS
	19/F	1997 Oct 25	3 2 3 3 3 2 5 3 2 5 1 4 3 2 2 5 2 2 1 3 4 4 6 3	700036777760771	91	X3	RRRRSSSRSRSXX
	35/F	1996 Dec 11	3 2 3 3 3 2 5 3 2 5 1 4 3 2 2 5 2 2 1 3 4 4 6 3	700036777760771	91	X3	RRRRSSSRSRXXX
	16/F	1996 Oct 17	3 2 3 3 3 2 5 3 2 5 1 4 3 2 2 5 2 2 1 3 4 4 7 3	700036777760771	91	X3	RRRRSSSRSSXXX
	28/F	1996 Oct 17	3 2 3 3 3 2 5 3 2 5 1 4 3 2 2 5 2 2 1 3 4 4 6 3	700036777760771	91	X3	RRRRRRSRSRSXX
439‡	21/M	2001 Oct 25	3 1 4 3 3 2 3 3 2 1 1 5 3 2 5 3 3 4 2 3 4 3 2 2	777777777460771	1905	T1	RRRRRRSRRRSSS
	19/M	2002 Jun 17	2 2 4 3 2 1 4 3 2 6 1 5 3 2 2 4 4 4 1 3 3 2 5 2	777777407560731	1355	T2	RRRRSSSSSRSSS
645	23/M	2001 Nov 13	2 1 4 3 3 2 4 2 2 6 1 5 3 2 1 4 3 4 1 5 3 4 4 2	777737607760771	93	LAM5	SRRSSSSSSRSSS
	22/F	2002 Jul 3	2 2 4 3 3 2 4 2 2 6 1 5 3 2 1 4 3 4 1 5 3 4 4 2	777737607760771	93	LAM5	SRRRSSSSSRSSS
	27/F	2001 Jan 24	2 1 4 3 3 2 4 2 2 6 1 5 3 2 1 4 3 4 1 5 3 3 4 2	777737607760771	93	LAM5	SRRRSSSSSRSSS
976‡	27/F	2002 Aug 27	4 1 4 3 2 2 3 3 2 1 1 5 3 2 4 3 3 4 2 3 4 3 2 2	777777777760771	53	T1	RRRRSSSSSRSSS
	26/M	2002 Mar 30	3 2 3 3 3 2 3 3 2 5 1 4 3 2 2 5 3 4 4 3 4 7 6 3	700036777760771	91	X3	RRRRSSSSRRRSS
977‡	19/M	2003 Feb 21	2 2 4 3 3 2 4 3 2 6 1 5 3 2 3 3 3 4 2 1 2 4 7 2	776177607660731	ND	ND	SRRRSSSSSRSSS
	46/F	NA	4 2 4 3 5 2 3 3 2 5 1 7 3 3 3 5 5 4 4 3 4 6 8 2	000000000003731	190	Beijing	SRRRSSSRRRSSS
	20/M	2002 Jul 5	2 2 4 3 3 2 4 3 2 6 1 5 3 2 3 3 3 4 2 1 2 3 7 2	776177607660731	ND	ND	RRRRSSSRSSSSS

*MDR TB, multidrug-resistant tuberculosis; ID, identification, MIRU-VNTR, mycobacterial interspersed repetitive unit–variable number tandem repeat; ST, shared type; ND, not determined because ST and lineage could not be assigned on the basis of spoligotype; NA, not available. †Antimicrobial drugs tested were streptomycin (S), isoniazid (H), rifampin (R), ethambutol (E), capreomycin (C), ciprofloxacin (F), cycloserine (Y), ethionamide (T), kanamycin (K), pyrazinamide (Z), amikacin (A), levofloxacin (L), and *p*-amino salicylic acid (P). Susceptibility patterns: S, susceptible; R, resistant; X, no result. ‡These 10 households were ultimately categorized as probable settings of multiple MDR TB introductions for subsequent analysis.

**Table A2:** Persons with >1 distinct MDR *Mycobacterium tuberculosis* isolate, Lima, Peru, 1996–2004*

House ID no.	Patient age, y/sex	Date	MIRU-VNTR pattern	Spoligotype	ST	Lineage	Drug susceptibility, SHRECFYTKZALP†
192	23/F	2001 May 31	2 1 5 3 3 2 3 1 2 4 1 2 2 2 1 4 3 4 1 5 3 3 8 2	777777607760731	60	LAM4	RRRRSSSRSRXSS
		2002 May 16	+ 2 4 3 3 2 4 3 2 6 1 3 3 2 1 4 3 4 2 1 2 + 6 2	576177607760771	1354	LAM3	RRRRSSSSSRSSS
439	19/M	2002 Apr 26	3 1 4 3 3 2 3 3 2 1 1 5 3 2 4 3 3 4 2 3 4 3 2 2	777777777460771	1905	T1	RRRRSSSSSRSSS
		2002 Jun 17	2 2 4 3 2 1 4 3 2 6 1 5 3 2 2 4 4 4 1 3 3 2 5 2	777777407560731	1355	T2	RRRRSSSSSRSSS
976	26/M	2000 Nov 28	4 2 4 3 2 2 3 3 2 1 1 5 3 2 4 3 3 4 2 3 4 3 2 2	777777777760771	53	T1	RRRRSSSRSRXSR
		2001 Jan 22	4 2 4 3 2 2 3 3 2 1 1 5 3 2 4 3 3 4 2 3 4 3 2 2	777777777760771	53	T1	SRRRSSSRSXXSR
		2001 Feb 2	4 2 4 3 2 2 3 3 2 1 1 5 3 2 4 3 3 4 2 3 4 3 2 2	777777777760771	53	T1	SRRRSSSSSXXSS
		2001 Jun 4	4 2 4 3 2 2 3 3 2 1 1 5 3 2 4 3 3 4 2 3 4 3 2 2	777777777760771	53	T1	SRRRSSSSSRXSS
		2002 Mar 30	3 2 3 3 3 2 3 3 2 5 1 4 3 2 2 5 3 4 4 3 4 7 6 3	700036777760771	91	X3	RRRRSSSSRRRSS
977	46/F	NA	4 2 4 3 5 2 3 3 2 5 1 7 3 3 3 5 5 4 4 3 4 6 8 2	000000000003731	190	Beijing	SRRRSSSRRRSSS
		2000 Apr 11	2 2 4 3 3 2 4 + 2 6 1 5 3 2 3 3 3 4 2 1 2 3 7 2	776177607660731	ND	ND	SRRRSSSSSSXXX
		2001 May 15	2 2 4 3 3 2 4 3 2 6 1 5 3 2 3 3 3 4 2 1 2 4 7 2	776177607660731	ND	ND	SRRRSSSSSSSSS
977	20/M	2001 Aug 13	2 2 4 3 3 2 4 3 2 6 1 5 3 2 3 3 3 4 2 1 2 3 7 2	776377617660731	ND	ND	SRRRSSSSSSSSS
		2001 Dec 3	2 2 4 3 3 2 4 3 2 6 1 5 3 2 3 3 3 4 2 1 2 3 7 2	776177607660731	ND	ND	SRRSSSSSSSSSS
		2002 Jul 5	2 2 4 3 3 2 4 3 2 6 1 5 3 2 3 3 3 4 2 1 2 3 7 2	776177607660731	ND	ND	RRRRSSSRSSSSS

*MDR, multidrug resistant; ID, identification, MIRU-VNTR, mycobacterial interspersed repetitive unit–variable number tandem repeat; ST, shared type; NA, not available; ND, not determined because ST and lineage could not be assigned on the basis of spoligotype. †Antimicrobial drugs tested were streptomycin (S), isoniazid (H), rifampin (R), ethambutol (E), capreomycin (C), ciprofloxacin (F), cycloserine (Y), ethionamide (T), kanamycin (K), pyrazinamide (Z), amikacin (A), levofloxacin (L), and *p*-amino salicylic acid (P). Susceptibility patterns: S, susceptible; R, resistant; X, no result.
